# Infantile and early childhood masturbation: Sex hormones and clinical profile

**DOI:** 10.4103/0256-4947.72271

**Published:** 2010

**Authors:** Heitham K. Ajlouni, Azhar S. Daoud, Saleh F. Ajlouni, Kamel M. Ajlouni

**Affiliations:** a From the Saint Michael’s Medical Center, Seton Hall University, New Jersey, USA; b From the National Center for Diabetes, Endocrinology and Genetics, Amman, Jordan; c From the King Hussein Medical Center, Jordan

## Abstract

**BACKGROUND AND OBJECTIVES:**

Few studies have explored the hormonal triggers for masturbation in infants and young children. Thus, we aimed to study the sex hormones and clinical profiles of masturbating infants and young children.

**METHODS:**

This case-control study involved infants and young children who masturbate and were referred to three pediatric neurology clinics between September 2004 and 2006 (n=13), and a similar control group. All children underwent basic laboratory investigations prior to referral. Other tests included electroencephalography (n=8) and brain neuroimaging (n=9). We measured dehydroepiandrosterone sulfate, 17-hydroxyprogesterone, free testosterone, estradiol, dehydroepiandrosterone, sex hormone-binding globulin (SHBG), and androstenedione in all participants.

**RESULT:**

The median age at the first incident was 19.5 months (range, 4-36 months); the median masturbation frequency, 4 times/day; and the median duration of each event, 3.9 min. The subjects masturbated in both prone (n=10) and supine positions (n=3); two subjects used the knee-chest position. All subjects showed facial flushing; 6, friction between the thighs; 5, sweating; 9, sleeping after the event; and 12, disturbance on interruption. EEG was abnormal in one of eight subjects tested, and neuroimages were normal in all of nine subjects examined. The case and control groups had comparable levels of all sex hormones, except estradiol, which showed significantly lower levels in the case group (*P*=.02).

**CONCLUSION:**

Masturbation in children seems to be associated with reduced estradiol levels, but not with other sex hormones. Further studies are needed to confirm our findings.

Childhood masturbation was reported by Still in 1909.^1^ It is characterized by self-stimulation of the genitalia frequently associated with unusual posture and movement, sweating, flushing, tachypnea, and typically begins in infancy and early childhood.^2^ Masturbation is a normal behavior in adolescence, occuring in 90% to 94% of males and 50% to 60% of females at some point in their lives; maturation of sex hormones predisposes to the activity.^3^–^5^ Previous reports on infants and early childhood masturbation are sparse with no attempts to identify the role of sex hormones in such a situation, despite the evidence that sex hormones are known to predispose to adolescentmasturbation behavior.^5^,^6^ In infants and young children, unusual postures and movements occur during masturbation and may be misdiagnosed as seizures, movement disorders, abdominal pain, colic, or other neurologic or medical problems.^7^–^10^ Extensive unwarranted investigations may be performed.^10^–^12^ To our knowledge, assessment of the levels of sex hormones as a possible predisposing factor has not been carried out before. The purpose of this study was to describe the clinical characteristics of masturbatory behaviors in 13 children referred to three different child neurology clinics in Jordan, to assess their sex hormones levels, and to compare these with that of a control group.

## METHODS

This was a prospective study of all infants and young children referred to the participating pediatric neurology clinics between September 2004 and 2006, diagnosed as having gratification disorder. A data collection sheet was developed which included information on demographic characteristics, a detailed history on the features of the movements during masturbation, clinical examination, neurodevelopment assessment, as well as the levels of sex hormones (dehydroepiandrosterone sulfate (DHEAS), 17-hydroxyprogesterone [17OHP], free testosterone, estradiol, dehydroepiandrosterone (DHEA), sex hormone-binding globulin [SHBG], and androstenedione). All hormone levels were determined using commercially available kits as follows: DHEAS and 17OHP were measured by RIA, DHGA by immunoradiometric assay (Immunotech Marseille, France), free testosterone by RIA (Biosource Europe S.A., Belgium), estradiol by a microparticle enzyme immunoassay using an AXsym machine (Abbott Labs, IL, USA), SHBG by an enzyme immunoassay using a Cobas machine (Roche Diagnostics, Mannheim, Germany), and androstenedione by an enzyme immunoassay (DSL, Dallas, TX, USA). To assess the role of sex hormones in this condition, 13 age- and sex-matched controls were selected from children attending the same clinics for reasons other than masturbation. Blood samples were obtained from all controls and assessed for sex hormones using the same techniques as for the cases. The mean levels of the sex hormones were compared between cases and controls using the two-sample independent t test. Other tests performed included EEG (n=8), brain CT scan (n=8), and brain MRI (n=1). Five of the children were wrongly diagnosed as having epilepsy and were on a maintenance antiepileptic drug treatment prior to referral to our clinics. Basic blood tests included complete blood count, serum electrolytes, and liver and kidney profiles. Echocardiography was performed on all children before the referral. The ethics committees of the three institutes approved the study protocol. A verbal consent was obtained from the parents of all participants.

## RESULTS

Thirteen infants and young children exhibiting masturbation were enrolled in this study. **Table 1** shows the characteristic features of events associated with masturbation. Antiepileptic drugs were given to five of the children, but did not have in any clinical benefit. A comparison of sex hormone levels between all cases and controls is shown in **Table 2**. **Table 3** shows the corresponding values in female cases. As shown in **Tables 2** and **3**, estradiol levels were found to be significantly lower in cases as compared to controls (*P*=.03). There was no difference in the levels of all other sex hormones between the two groups. The mean estradiol level was also found to be lower in male cases (9.3 pg/mL) as compared to male controls (14.3 pg/mL) (not reported in the tables), but the difference was not statistically significant (*P*=.50), possibly due to the small sample size.

**Table 1:** Characteristic features of events associated with masturbation in 13 Jordanian children.

Patient	Sex	Age of onset	Color change	Posture	Duration	Frequency
1	F	6 mo	Flushing	Prone, supine	5 Min	3/day
2	F	6 mo	Flushing	Knee-chest	2 Min	20/day
3	M	18 mo	Flushing	Prone	5 Min	2/day
4	M	36 mo	Flushing	Prone	5 Min	3/day
5	F	12 mo	Flushing	Friction of thighs	5 Min	5/day
6	M	36 mo	Flushing	Prone	5 Min	2/day
7	F	36 mo	Flushing	Prone	10 Min	2/day
8	F	42 mo	Flushing	Supine	3 Min	3/day
9	F	13 mo	Flushing	Prone	3 Min	3/day
10	F	20 mo	Flushing	Prone	4 Min	4/day
11	F	18 mo	Flushing	Prone	5 Min	6/day
12	F	4 mo	Flushing	Prone, knee-chest	4 Min	10/day
13	F	12 mo	Flushing	Prone	5 Min	3/day

**Table 2:** Hormonal profile of children exhibiting masturbation.

	Status	N	Mean	SD	*P*
Age (years)	Control	13	2.02	0.91	.15
	Case	13	2.67	1.28	
DHEAS (μg/dL)	Control	13	6.59	12.54	.54
	Case	13	4.37	3.43	
17OHP (nmol/L)	Control	13	2.83	2.29	.75
	Case	13	2.55	1.94	
Free testosterone (pg/mL)	Control	13	0.31	0.14	.87
	Case	13	0.31	0.15	
17-estradiol (pg/mL)	Control	13	19.46	8.50	.03
	Case	13	12.31	6.73	
DHEA (ng/mL)	Control	13	1.28	0.97	.89
	Case	13	1.23	0.67	
SHBG (nmol/L)	Control	13	71.43	24.00	.20
	Case	13	86.07	32.27	
Androstenedione (ng/mL)	Control	13	0.14	0.16	.95
	Case	13	0.15	0.19	

**Table 3:** Hormonal profile of female children exhibiting masturbation, Jordan 2009.

	Status	N	Mean	SD	*P*
Age (years)	Control	10	1.78	0.86	.27
	Case	10	2.34	1.19	
DHEAS (μg/dL)	Control	10	2.27	1.74	.12
	Case	10	4.54	3.80	
17OHP (nmol/L)	Control	10	3.39	2.55	.56
	Case	10	2.77	2.04	
Free testosterone (pg/mL)	Control	10	0.28	0.12	.40
	Case	10	0.34	0.16	
17-estradiol (pg/ml)	Control	10	21.78	6.38	.02
	Case	10	13.20	7.51	
DHEA (ng/ml)	Control	10	0.97	0.30	.24
	Case	10	1.29	0.74	
SHBG (nmol/l)	Control	10	72.88	27.82	.52
	Case	10	82.42	34.54	
Androstenedione (ng/ml)	Control	10	0.16	0.17	.94
	Case	10	0.16	0.21	

## DISCUSSION

Masturbation is considered to be a common normal behavior in adolescents. The physiological and hormonal changes that occur during such activity have been well-documented.^5^ However, in infants and young children, masturbation can be difficult to recognize due to the absence of genital manipulation, as well as the variable manifestations of this behavior.^3^ Childhood masturbation, if unrecognized, may lead to considerable parental anxiety, unnecessary investigations, and inappropriate and potentially harmful therapies.^2^–^12^ The paroxysmal tightening of the thighs, rocking pelvic movements or other rhythmic activities, mechanical pressure applied to the supra-pubic area, grunting, facial flushing, irregular breathing, and sweating during the event, may be misinterpreted as abdominal pain, urinary symptoms, or epileptic seizures.^8^–^12^ In our subjects, masturbation was previously misdiagnosed in many of the cases as a seizure disorder, dystonia, or abdominal pain, resulting in extensive diagnostic testing in the majority of our children, as well as initiation of many unnecessary medications. This demonstrates that even for an experienced movement disorder specialist, the distinction between paroxysmal movement disorders and masturbatory behavior can be difficult to make,^10^ if the fact that these infants and young children are responsive to all stimuli during masturbation is missed. In a previous study, 8 of 12 patients with similar characteristics had been treated with different antiepileptic medications.^3^

Our diagnosis was made on the basis of the Fleisher and Morrison study^9^ that reported the frequency of an event to vary from 1/week to 12/day, with a mean frequency of 16/week, and a median of 7/week. The mean duration of the event was 9 minutes (median 2.5 minutes, range 30 seconds to 2 hours).^9^ The median frequency of events in our study was 4/day, and the median duration of the event was 3.9 minutes. The female-to-male ratio was 3:1 in our study. Varied ratios have been reported in other studies.^6^,^9^,^13^ Consistent with our findings, masturbation has been reported to start in most children before 2 years of age.^6^ Since Jordan is a sexually conservative country with no formal sex education, childhood masturbation may create more parental concern than in Western societies, and the referral rate may differ. Ten (77%) of our children did not attend any follow-up visits after their parents were informed about the diagnosis of childhood masturbation, possibly due to the concern of stigmatization. The etiology of childhood masturbation and its predisposing factors are still controversial and poorly understood. Childhood masturbation has been linked to emotional deprivation, which may in turn lead to more self-stimulation.^14^ It may also be associated with sexual abuse.^14^ A possible correlation of childhood masturbation to the duration of breast-feeding has also been reported: masturbation was found to be significantly associated with weaning, but not with pacifier usage.^6^

To our knowledge, this is the first study to examine the role of sex hormones in masturbation behavior in children. The finding of a significantly lower level of estradiol in cases as compared to controls is interesting, but should be interpreted with caution. Further studies need to be conducted to conform our results.
